# Farming Related Trauma Injuries in Southern West Virginia With a Focus on Risks, Injury Trends, and Associated Co-morbidities

**DOI:** 10.7759/cureus.6031

**Published:** 2019-10-30

**Authors:** Ravi Viradia, Frank H Annie, Maher Kali, John D Hayes, Frederic Pollock

**Affiliations:** 1 Surgery, Charleston Area Medical Center/ West Virginia University, Charleston, USA; 2 Cardiology, Charleston Area Medical Center, Charleston, USA; 3 Clinical Research, Charleston Area Medical Health Education and Research Institute, Charleston, USA; 4 Surgery, Charleston Area Medical Center, Charleston, USA

**Keywords:** farming, injuries

## Abstract

Background

The implementation of safety policies in farming-related injuries in West Virginia has been lacking. Farming-related injuries have resulted in massive injuries that have resulted in life long injuries and death. Therefore, this study aims to review 12 years of our level 1 trauma data and describe the incidence rate and patterns of priority-related farming injuries in West Virginia, as well as the specific co-morbidities and related injuries that might be more susceptible to damage.

Methods

We examined 82 cases of farm-related injuries that required trauma-priority related intervention from 2005 -2016. We harvested data from the Charleston Area Medical Center Trauma registry to investigate associated injuries. We defined farm equipment as any mechanical or automated tool used on a farm for related farm upkeep or farm-related activity. Multinomial logistic regression was used to understand the overall impact on the differing effects of years of injuries.

Results

The total number of farming-related injury cases was 82. The most statistically suggestive finding was those that had a positive narcotics urine test at (p= 0.062) (-.3230-12.82). Those with a history of CHF (congestive heart failure) also had a significant statistical relationship at (p=0.001) (-5.477-1.394). Alcohol use disorder was also a significant statistical relationship (p=0.012) (-5.127--.6728). The most common injuries were concussions at 18 % ( 15/82) followed by rib fractures at 17 % ( 14/82).

Conclusion

Farming-related injuries appear to have increased risks on specific body and organ systems, as described in our initial data analysis. Specific co-morbidities also have been documented to show a higher risk of injury and would need further investigation. Specific years show a higher prevalence of farming injuries compared to other years. Further research is needed to explore these underlying findings.

## Introduction

The implementation of safety policies in farming-related injuries in West Virginia has been lacking. Farming-related injuries have resulted in massive injuries that have resulted in life long injuries and death. Therefore, this study aims to review 12 years of our level 1 trauma data and describe the incidence rate and patterns of priority-related farming injuries in West Virginia farmers, as well as the specific co-morbidities and related injuries that might be more susceptible to damage [1]. Farming tools evolved over the years from planting by using bare hands to sculpture a stony cultivating tool [2-7]. The rapid growth of the world population and the increasing food demand lead to the use of fertilizers and chemicals to enhance faster corps production.

Moreover, farming came along with animal domestication for different purposes, from pulling a plow and transporting harvest, in addition to raising cattle and different kinds of farm animals for the meat and milk. Moreover, the farming culture involved every family member, young or old male or female in helping on the field. Thus, dealing with such a complex environment lead to different types of injuries from operating a machine or contact with an animal and even poisoning from chemical exposure, and affected all ages and genders [8-16]. The state of West Virginia has farming communities from small family-run farms to mid and large scale industrial crops producing farms; however, the implementation of safety policies in farming-related injuries in West Virginia has been lacking. Farming-related injuries have resulted in massive injuries that have resulted in life long injuries and death. Therefore, this study aims to review 12 years of our level 1 trauma data and describe the incidence and patterns of priority-related farming injuries in our tertiary medical center in southern West Virginia, as well as the specific co-morbidities and related injuries of the body that might be more susceptible to damage. 

## Materials and methods

We examined 82 cases of farm-related injuries that required trauma-priority related intervention from 2005 -2016. We harvested data from the Charleston Area Medical Center Trauma registry to investigate associated injuries. We defined farm equipment as any mechanical or tool used on a farm for related farm upkeep or farm-related activity. Multinomial logistic regression was used to understand the overall impact on the differing effects of years of injuries.

## Results

**Figure 1:**
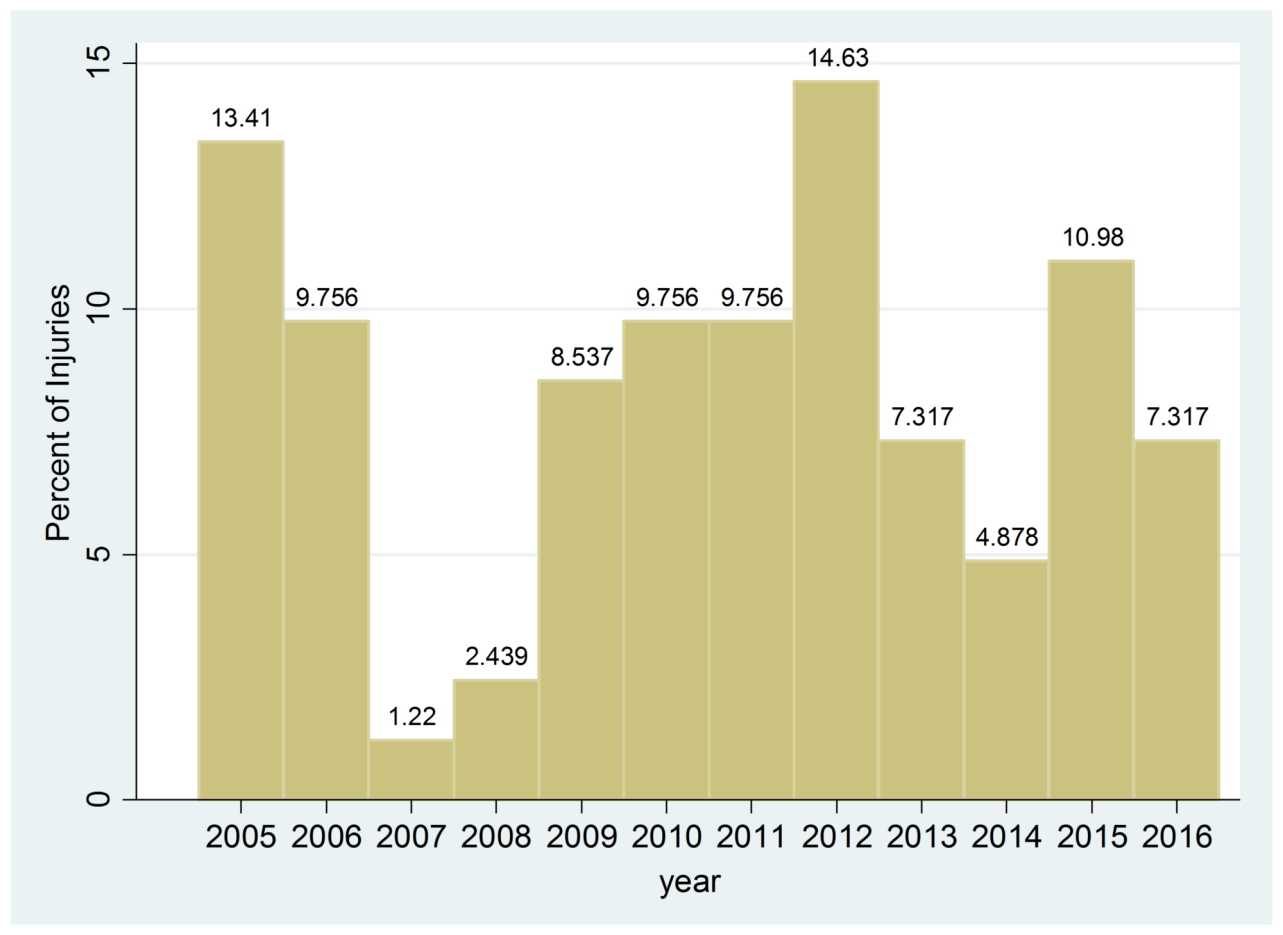
Injuries Through Time

The variables collected are as follows: The sample size representing the total number of farming-related injury cases was 82 (Table 1). The average age was 60.8 years. The most common injuries were concussions at 18%-(15/82) followed by rib fractures at 17%-(14/82). In regards to 2005-2018 injury, the highest was in 2012 at 14.63 % while the lowest was in 2007 at 1.22% (figure 1). The most statistically suggestive finding had a positive narcotics urine test at (p= 0.062) (-.3230-12.82). Those with a history of CHF also had a significant statistical relationship (p=0.001) (-5.477-1.394). Alcohol use disorder was also a significant statistical relationship (p=0.012)(-5.127--.6728). 

**Table 1:** Injuries

Injuries	N = 82
Other Injury	5
Amputation At Above Knee	2
2^nd^-Degree Burns	1
Metacarpal Fracture	1
Cerebral Concussion	15
Cerebral Hematoma	7
Cervical	2
Crush Hand	1
Spine Fracture	3
Distal Radius	2
Distal Tibia	0
Fracture	5
Finger Amputation	3
Ribs Fracture	14
Lateral Fracture	1
Lumber Injury	3
Lung	1
Face Injury	1
Metatarsal Fracture	2
Pelvic	8
Skin	3
Finger Fracture	2

 

## Discussion

Farming related injuries in southern WV have been prevalent, and our retrospective data from our trauma registry has suggested that multiple organ systems are at risk of injury. Farming itself is a dangerous profession, and safety regulations in the occupation and the operating equipment involved are not as stringent as other specialties. Peek-Asa reported close to 10% of farmers get injured while working on the farm and 1769 death in Canada between 1990 and 2005, including children [1-7]. Our sample size consisted of farmers who were injured on a farm and were taken to our level 1 trauma center for further evaluation and work up. Again, we defined farm equipment as any mechanical or tool used on a farm for related farm upkeep or farm-related activity [3-5}. We found that the most common farming injury sustained was a concussion, followed by rib fractures and pelvic fractures. We also found that narcotic usage, congestive heart failure, and positive alcohol were statistically significant for an increase in injury. We also took our evaluation of data a step further to track the injury trends of the incidence during each year from 2005-2016. Our analysis found that 2012 to be the highest year with farming injuries presenting to our level 1 trauma center. 

## Conclusions

Farming-related injuries appear to have increased risks on specific body and organ systems, as described in our initial data analysis. Specific co-morbidities also have been established that show a higher risk of injury and would need further investigation. Specific years show a higher prevalence of farming injuries compared to other years. New research is required to explore these underlying findings. Farming-related injuries appear to have increased risks on specific body and organ systems, as described in our initial data analysis. Additionally, understanding the type of injuries will help the clinician to be better prepared to manage such injuries in rural medical centers. This study promotes awareness of the need for developing better public health occupational policies and preventive teaching programs to reduce these preventable injuries. 
